# Feasibility and outcomes of an out-of-school and home-based obesity prevention pilot study for rural children on an American Indian reservation

**DOI:** 10.1186/s40814-018-0322-4

**Published:** 2018-07-26

**Authors:** Blakely Brown, Kari Jo Harris, Daniel Heil, Michael Tryon, Aric Cooksley, Erin Semmens, James Davis, Krupa Gandhi

**Affiliations:** 1 Department of Health and Human Performance, The University of Montana, McGill Hall 206, 32 Campus Drive Missoula, Missoula, MT 59812 USA; 2 School of Public and Community Health Sciences, University of Montana, 32 Campus Drive, Missoula, MT 59812 USA; 3 Department Health and Human Development, Montana State University, 210 Romney Gym, Bozeman, MT 59715 USA; 4 Polson Medical Fitness Center, Summit Medical Fitness Center, 205 Sunnyview Lane, Kalispell, MT 59901 USA; 5 Boys and Girls Club of the Flathead Reseration and Lake County, 63055 US-93, Ronan, MT 59864 USA; 6 Department of Complementary and Integrative Medicine, University of Hawaii John A. Burns School of Medicine, Biosciences Building Suite 211, 651 Ilalo Street, Honolulu, HI 96813 USA; 7 Department of Pharmacology and Experimental Therapeutics, Division of Biostatistics, Thomas Jefferson University, 1015 Chestnut Street, Suite 520, Philadelphia, PA 19107 USA

**Keywords:** Child health behavior, Rural, Native American, Obesity prevention, Parents, Out-of-school settings, Intervention feasibility

## Abstract

**Background:**

Children living in rural areas are at higher risk for obesity compared to urban children, and Native American (NA) children have the highest prevalence of overweight/obesity for all races combined. Out-of-school programs (OOSPs) are a promising setting to improve children’s health. Parents are important in supporting their child’s obesity-related behaviors, yet it remains unclear what combination and dose of parent engagement strategies is feasible and optimal. This study’s primary objective was to assess the feasibility of an OOSP and home-based obesity prevention intervention for rural NA and non-NA children.

**Methods:**

This was an 11-week, two group, randomized feasibility study. Participants were children and their parents at one OOSP on a rural American Indian reservation. Children, ages 6–9, were randomized to receive the Generations Health (GH) intervention or comparison condition. The GH group received daily activities focused on physical activity (PA), nutrition, sleep, and reducing TV/screen time, and frequently engaged parents. The comparison group received usual OOSP activities. To assess intervention feasibility, we measured recruitment and participation rates and program satisfaction. We assessed pre- to posttest changes in body composition, PA and sleep patterns, dietary intake and Healthy Eating Index-2010 (HEI-2010) scores, TV/screen time, and nutrition knowledge. We report recruitment and participation rates as percentages and participants’ program satisfaction as means. Two-tailed paired *t* tests and 95% confidence intervals were used to detect changes in behavioral and health outcome variables.

**Results:**

Forty-six children met age eligibility criteria; following screening, 52% (24/46) met the inclusion criteria and 96% (23/24) were randomized to the study. Overall, 91% of the children participated in the intervention and 100% participated in at least some of the posttest assessments. Parents reported high program satisfaction (mean rating of 4, on a 1–5 scale). Our outcome measure for child adiposity, zBMI, was reduced by 0.15 in the GH group, but increased by 0.13 in the comparison condition. Meaningful changes were evident for total kilocalories, HEI-2010 scores, PA, TV/screen time, and nutrition knowledge.

**Conclusions:**

High recruitment, participation and program satisfaction and positive health and behavioral outcomes at 11 weeks provide encouraging indications of the feasibility and potential effectiveness of the intervention.

**Trial registration:**

ISRCTN24274245

## Background

The nearly 12 million children living in rural areas in the USA have 26% greater odds of obesity compared to urban children [1], even after controlling for sociodemographics [2–5]. Rural residency is also often compounded by minority ethnicity to further increase obesity risk [2, 5–7]. Recent studies suggest that preadolescent children ages 6–11 years are most likely to experience acceleration of weight gain during childhood [8], suggesting it may be especially important to provide effective health promotion programs during the early elementary school years. Obesity prevention trials tend to target preschool (i.e., ages 3–5) [9] or older elementary and middle-school (i.e., ages 10–14) [10] children, leaving the younger elementary school children (i.e., ages 6–9) understudied. A 2011 Cochrane report identified promising strategies for reducing or stabilizing body mass index (BMI) in elementary-aged children. Strategies involved using a multilevel approach to change cultural practices to better support children eating healthier foods, parent support and engagement, and home activities that encouraged activity, healthy eating, and less screen time [11]. A recent review of obesity disparities research targeting children and minority populations in underserved communities shows that very few trials intervene at multiple levels or intervene on multiple factors (e.g., home, school, afterschool, local organization policies) [12, 13]. Further, most obesity prevention interventions focus only on one context—schools. A recent multilevel intervention that improved fruit and vegetable consumption in mostly Black and Hispanic children living in Southern and Pacific Coast rural communities [14] shows that a multilevel approach can be successfully implemented in diverse rural communities, but implementation and rigorous evaluation of such interventions is rare.

Poverty is particularly acute among rural children, and residents often lack access to healthy foods and physical activity (PA) opportunities compared with urban residents. Despite these challenges, rural residents often have strong social ties and common values for well-being and health [15, 16]. Community solutions to address food access barriers in rural communities have included collaborative strategies to increase availability of healthful food through traditional and non-traditional food sources [17]. There are often fewer competing activities (e.g., music or art events) in rural communities, thus increasing the potency and interest in family-based activities. Leveraging the strengths unique to rural communities may ensure responsive and effective strategies to improve child health in rural environments [18].

Whereas multi-level and multi-component interventions are necessary, it is critical to have a setting and context in which many rural children and families are engaged to be the epicenter to launch the intervention. With more than 10 million children participating every year, out-of-school programs (OOSPs) like Boys and Girls Clubs provide a promising setting for obesity prevention efforts without competing for academic time in school [19–23]. OOSPs serve a high proportion low-income, rural children and children of color [24] and serve snacks and meals guided by national standards [25]. Compared to health promotion studies with schools as the primary place for the intervention components, less work has been done with OOSPs as the initial implementation point [26]. Two studies evaluating the feasibility of implementing obesity prevention activities for children in OOSP Club settings in non-rural (e.g., urban) areas [27, 28] showed that structured physical activity, nutrition education, and family components can be incorporated into Club programs, that family nights offered ways for families to be involved in what children were doing outside of school [28], and that the majority of children (70%; *n* = 371) in the study can complete annual BMI assessments [27]. Both studies reported success in implementing training sessions for staff delivering the interventions, and providing promising frameworks to support the capacity of OOSPs to create healthy environments [27, 28]. While these outcomes suggest strong feasibility for implementing obesity prevention strategies in urban OOSP settings, to our knowledge, there are no child obesity prevention studies in the literature in *rural* OOSP settings with a high proportion of Native Americans. Further, very few OOSP interventions have extended beyond the OOSP in order to have the most substantial and sustainable efficacy.

In working with children to improve healthy lifestyles, it is necessary to engage their parents. However, many researchers note that strategies to engage parents to make changes to obesogenic environments are still underdeveloped [29]. Children lack cognitive maturity to generalize behaviors in one setting (i.e., OOSP) to other settings, and parents provide access to most eating and activity opportunities [30, 31].

The Ecological Model of Physical Activity (EMPA) [32, 33], originally designed to understand PA, can be applied to other behaviors and posits that physical and social environmental factors, processes, and linkages directly and indirectly impact behavior change and maintenance [33, 34]. The EMPA further suggests that events and behavior choices are dynamic: things that happen in one setting to one person can influence other people in the same setting (a meso-level environmental linkage) or those in another unrelated setting (an exo-level environmental linkage) sometimes called a “ripple effect” [35].

This study assessed the feasibility and behavioral and health-related outcomes of the Generations Health intervention in an 11-week, 48-session randomized, controlled pilot study implemented in a rural, OOSP Club on a rural, American Indian (AI) reservation. Knowledge of this information sets the foundation for future effectiveness studies and subsequent efforts to scale up and extend the reach of the intervention in rural communities with a high proportion of Native Americans.

## Methods

### Generations health intervention

The Generations Health program components promote dynamic, integrated activities for increasing PA, reducing TV/screen time, improving sleep, preferring and consuming healthy foods in children, and engaging parents to support these behaviors in their child (Table 1).

**Table 1:** Generations Health components and dose

Intervention components	Dose
40 min MVPA, 20 min eating healthy foods and fewer kcals, sleep and limiting screen/TV time to children at the OOSP	1 h, 3 times/week
Text messages to parents	Once per week
Toolkits given to parents at pick up time at the OOSP that contain resources to support integrated home-based nutrition, PA, sleep and limiting screen/TV time activities	3 times/week
Interactive family nights for parents, child and family members	2 h/once per month
Information meetings about the study for parents	1 h, once during the study

*MVPA* moderate to vigorous activity, *OOSP* out-of-school program site

Each Generations Health OOSP session begins with 40 min of moderate-to vigorous physical activity (MVPA) that is followed by 20 min of nutrition, reducing sedentary behaviors including TV/screen time, and improving sleep activities. The intervention targets for nutrition and PA are based on previous studies in OOSP settings [20, 36–41], our previous pilot study [42], and national recommendations [43, 44]. Project staff encourage children to participate in the Generations Health activities at least three times per week on days of their choice while they attend the OOSP.

The *physical activity portion of the program* is designed to engage children in activities such as aerobic dance, jump rope, hula-hoop, and kickball by increasing their activity in incremental steps and in ways that are intended to be enjoyable. All activities are game-like and non-competitive. These strategies are designed to increase a child’s sense of self-efficacy and keep children active at a moderate to vigorous level. The *nutrition portion of the program* is designed to build skills that make it easy and fun to consume a diet that delivers a balanced array of nutrients from fruits and vegetables, whole grains, lean meats, legumes, and foods and beverages low in sodium and added sugars. Sessions use hands-on activities for understanding portion size, calorie and nutrient content on food labels, and limiting unhealthy snacks. *The sleep portion of the program* is designed to improve sleep by having children role play bedtime routines and engage in stories and conversations about sleep. The *reducing sedentary behaviors portion* of the program is designed to reduce the amount of time children spend watching TV or screens. Sessions use games that challenge children to turn off TVs and electronic devices and play active games instead, and hands on activities comparing heart rates during sedentary and active behaviors with discussions of the differences.

Parents of children in the Generations Health program receive take-home toolkit materials and activities three times per week from program staff when they pick up their child(ren) at the OOSP. Each toolkit contains interactive, hands-on learning activities and healthy lifestyle reinforcers such as cooking supplies (e.g., coolers with foods their child made during the program for the family to taste at home, recipes, food coupons, fruit challenge games, family activities that repeat concepts from OOSP sessions), exercise equipment (e.g., jump ropes with jumping songs, flex bands and exercises), bedtime routine activities (e.g., stories about children living in rural areas, bedtime routine check-off list games) and turn off the TV/screen time interactive challenge activities. Parents also participate in monthly family nights. Each family night begins with families eating a healthy dinner together (i.e., stew made with bison meat and vegetables and salad). Then families rotate through stations where they engage in dynamic hands-on activities that repeat concepts from the Generations Health sessions. For example, at a “Make a tasty snack” station, families select from raw nuts and dried fruit to make a healthy trail-mix snack. At a “Family fun games!” station, families play a relay race and giant tic tac toe games. At a “Is there caffeine in there?” station, families create a list of non-caffeinated drinks that can be enjoyed closer to bedtime.

Many of the Generations Health activities are tailored to be culturally and contextually relevant for children and families living in the Northern Plains rural communities (Table 2). To tailor the activities, we conducted two focus groups and eight interviews with parents and OOSP staff in two communities on the AI reservation [45, 46]. All sessions were digitally recorded. Focus group length ranged from 60 to 90 min, while interview session length ranged from 30 to 60 min. Moderators and interviewers took detailed notes during each session, and reviewed and clarified the written notes and summarized the comments by topic area (PA, healthy eating, parent-child engagement, culture) immediately following each session. These data indicated that intervention activities should include preparing and eating wild game, horseback riding, dancing, and Native American traditional games like Shinny, using a group format and interactive activities [47] and encompass more diverse cultures of rural areas; community members who might not only be genetically white or Native American, but also have Scottish, Latino, or Irish ancestry [45]. For example, during the intervention’s family nights, participants could taste Irish stew prepared using healthy ingredients and learn how to make the stew at home. Participants suggested activities like polka, square dance, drumming, and international games like lacrosse. Parents also said they were interested in intervention components targeting sleep, like bedtime routine check off lists. Because many rural families travel long distances, participants suggested car activities, like playing the 20 questions game to guess a healthy food. We also learned that parents were motivated to make broader changes in their children’s food and PA environments, and wanted to work with other parents to make these changes [46].

**Table 2:** Examples: cultural and contextual tailored intervention components at OOSP site

Activity	Description
Shinny game	Children play traditional game of Northern Plains Indian tribes
Eagles and Salmon game	Children play tag game as birds (eagles) and fish (salmon) in Northern Plains rural areas
Macarena and Mexican Hat dances	Children participate in Latin dances that are culturally relevant to the Latino/Hispanic populations living in rural areas
Traditional proteins	Participants taste traditional proteins from the Northern Plains region (e.g., dried bison, venison, pemmican) and play a guessing game about proteins from other cultures living in this region (e.g., Scotland, Brazil, Ireland)
Family nights	Families eat foods from other countries and Native American Tribes then take home recipes and ingredients to prepare at home

### Study design and setting

This was a randomized, pretest to posttest two-group feasibility study of the Generations Health intervention. The study was 11 weeks in duration and there were two data collection periods: baseline (September 2015) and final assessment (December 2015); data were analyzed in April 2016. The study took place at an existing Boys and Girls Club OOSP site in a small, rural town on an AI reservation (population 29,000) comprised of 33% NA and 66% non-NA people [48]. We worked with the Club Director and staff to target our recruitment strategies to this OOSP.

### Participants

We included Native American and non-Native American children at the OOSP site, age 6–9 years, and their parent, caregiver or guardian (from this point on referred to as parent) who made most of the food and activity decisions in the household were recruited to participate in the pilot study. Some of the parents had participated in the focus groups or interviews described earlier. Eligibility criteria for the children included being 6–9 years old, attending the OOSP at least 3 times per week, and planned to attend the OOSP throughout the study period. We focused on the 6–9 years old age group because they comprise most of the children regularly attending OOSPs [49] and the literature shows a paucity of interventions targeting 1st, 2nd, and 3rd graders. The OOSP accommodates children living with a physical disability. These children were not excluded from participating in the study. This study was approved by an Institutional Review Board at the tribal college on the AI reservation. All parent(s) provided written informed consent for their self, and for their child to participate and all children provided verbal and written assent to participate.

### Recruitment

In mid-August 2015, we obtained a list of age eligible children from the Club Director. Then, trained project staff recruited participants ages 6–9 by asking children if they wanted to participate, asking parents if they wanted their child to participate and giving them an informational letter about the study and advertising with fliers at the OOSP site. For families with more than one child in the eligible age range, one child was randomly selected as eligible to participate in the study.

### Baseline assessment

Participants completed all baseline assessments over a 15-day period from September 1–15, 2015. Children underwent a 28–45 min in person assessment (including the 24-h dietary recall and placement of an accelerometer by trained staff) and took home instructions about the accelerometer. Parents underwent a 12–45 min in person assessment (including attending their child’s dietary recall assessment).

### Randomization

Randomization to the Generations Health group or the usual OOSP activities group (from this point on referred to as the comparison group) occurred after parents and children completed all baseline measures. Project staff opened an envelope that contained pre-determined group assignments (Generations Health or comparison group); families were assigned to the group indicated in the envelope. Immediately following randomization, participants were handed a letter informing them of their group assignment and describing the activities associated with that group. To assure equal allocation, the randomization scheme was constructed using permuted block sizes of 4. Only the Co-Investigator (Harris) had access to allocation codes before randomization. As this was a feasibility study, no stratification was used.

To enhance feasibility and generalizability, eligible siblings who shared the same household were allowed to participate in the OOSP-site elements of the study except the outcome measurements. Brothers and/or sisters who were not eligible, but who had a sibling in the Generations Health condition, were encouraged to attend the monthly family nights. Study outcome measures were only collected from the sibling randomized and these were included in the data analysis. It was not possible to mask staff from knowing which group children were assigned. To compensate families for the time required for participation, after completing the study, families in the Generations Health group were paid $150 and families in the comparison group were paid $70. Parents received an additional $20 after completing each dietary recall interview with their child.

### Intervention group

Generations Health group activities for children were offered every day the OOSP was in session (48 days) over an 11-week period. These activities took place at a different location than the OOSP and children took a 5-min van ride to this location. Each week parents received three take-home toolkits (*n* = 33) with associated materials (e.g., coolers with recipe ingredients, strength and flexibility bands, pedometers) when they picked up their child(ren) at the OOSP. In addition, project staff hosted three family nights for all family members in this group, and a 1-h informational session during the first 2 weeks of the study. At this session, project staff talked more in-depth with parents of children in this group about the various intervention components such as the take-home toolkits, returning toolkit prize tickets to the OOSP, reminding parents of upcoming schedules for family nights and measurement outcomes, and answered questions about the study. Project staff sent parents text messages to remind them of key events, such as upcoming family nights. Project staff were existing employees of the OOSP, a tribal college student, tribal college community health and development staff, and a university graduate student. All project staff (*n* = 5) attended a day-long training session on delivering the Generations Health components 3 weeks before the study began.

### Comparison group

Children in the comparison group received the usual Club activities at the OOSP site, which routinely included opportunities for physical activity. There were no activities for parents in the comparison group, except to complete pre- and posttest measures.

#### Measures

##### Intervention feasibility—primary outcome measures

To assess intervention feasibility, project staff recorded recruitment and intervention and measurement participation information throughout the study. Also, project staff asked children to indicate that they completed a home activity by returning a tear-off portion of cards that contained instructions for the activity. The tear-off portion required a parent signature to indicate that the family participated in the activity. At the end of the study, parents rated their overall satisfaction with the Generations Health program on Likert scale ranging from 1 (not at all) to 5 (very high) and responded to open ended questions about the “best part” and “recommended changes” to the project. Similarly, for each family night, parents rated their likelihood of participating in similar events in the future on a Likert scale ranging from 1 (not at all) to 5 (very high).

##### Behavioral and health outcomes—secondary measures

To evaluate likely effectiveness, pre- and posttest measures included child adiposity measures [staff measured height (using a portable stadiometer, SECA 214) and weight (using an electronic scale, SECA 803)], child PA and sleep efficiency [(7-day wrist-worn activity monitors (AM)], child kilocalories (kcals) consumed, and HEI scores (parent and staff assisted 24-h dietary recall using the online National Cancer Institute’s ASA24™-2014) [50]. Child self-reported TV/screen time was measured by three questions that asked children to report the number of hours per day they spent watching TV, playing video games, and using a computer for non-school related activities. Responses were summed across the three questions to estimate the number of hours screen time viewed per day across a 7-day period. Children’s knowledge of nutrition was assessed by asking children to indicate which of 12 paired foods are “better for your health” [51]. Number of correct responses were summed and ranged from 0 to 12. The Healthy Eating Index-2010 (HEI-2010) is a measure of diet quality, independent of quantity that can be used to assess compliance with the US Dietary Guidelines for Americans and monitor changes in dietary patterns [52]. The HEI-2010 has 12 components including total fruit, whole fruit, total vegetable, greens and beans, whole grains, diary, total protein foods, seafood and plant proteins, fatty acids, refined grains, sodium, and empty calories. The maximum total HEI-2010 score is 100 [53].

##### Demographics

To describe the participants’ characteristics, at baseline parents completed a demographic questionnaire for themselves and their child, reporting ages, sex, ethnicity, annual family income, qualification for free or reduced school lunch, and food insecurity. Risk for food insecurity was measured by an affirmative response to either of the two items on the screener to identify families at risk for food insecurity [54].

##### Statistical analysis

Data from all parents and children were included in the analyses regardless of their level of participation in the intervention. We did not conduct a formal sample size calculation for this feasibility study; rather, we aimed to recruit sufficient participants to generate estimates of variability for our outcome measures and to generate preliminary estimates of effect for the intervention. We described participant characteristics using mean and standard deviations. To address intervention feasibility, we calculated recruitment and participation rates in the intervention activities and the outcome measures and report percentage; we also report participants’ program satisfaction as means. For the behavioral and health outcome variables, we used two-tailed paired *t* tests to detect changes in baseline (pretest) and end-of-treatment (posttest, 11 weeks after baseline) in child self-report assessment and child adiposity measures presented with a 95% confidence interval. These analyses were performed in SPSS 22.0 (SPSS, Inc., Chicago, IL) and STAT MP v15.0 (College Station, TX). The AM-derived PA, sleep efficiency and wake-after-sleep-onset data were evaluated using a multivariate repeated measures analysis of variance using Statistix 10; Analytical Software, Tallahassee, FL. Statistical significance was set at the 0.05 alpha level.

## Results

Fifty-two percent of the children were Native American, with a mean age of 8 years. Eleven (48%) had a BMI-for-age classification of normal weight and the rest were classified as overweight (13%) or obese (39%). All parents reported having a high school degree and most (74%) had completed at least some college. Twenty-six percent of the parents (6/23) were male. Overall, 82.6% qualified for the free and reduced school lunch program and 35% were at-risk for food insecurity. We were able to utilize all outcome measurement instruments initially proposed.

### Intervention feasibility

#### Recruitment

**Fig. 1:**
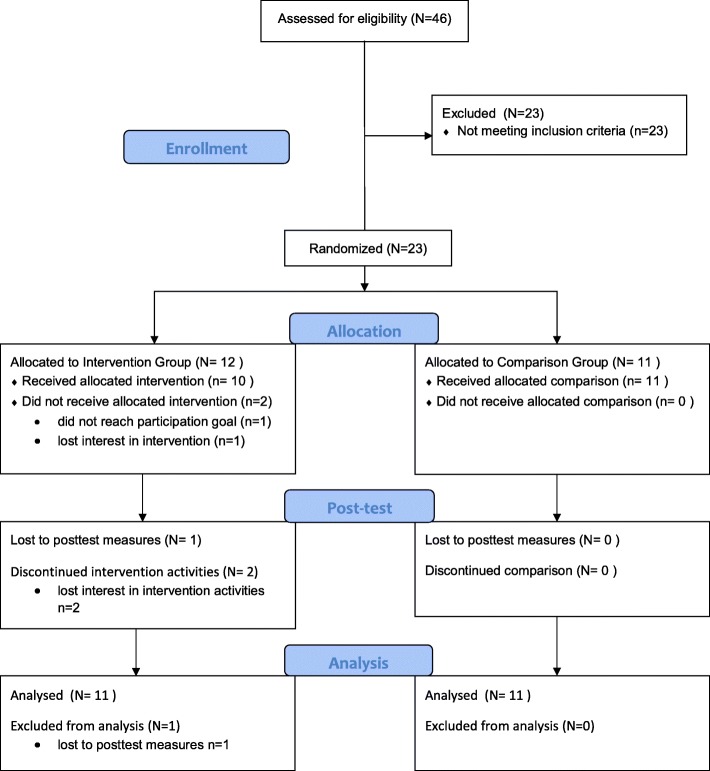
CONSORT 2010 flow diagram for the Generations Health Feasibility study

To begin recruitment for the study, the project’s lead investigator obtained a list of names of children enrolled in the Club who were 6 to 9 years old from the Club Director; 46 children met these initial eligibility requirements (e.g., age, enrolled in OOSP). Following further screening by a project staff member, 23 dyads of children and parents met final eligibility criteria that included the child being 6–9 years old and attending the Club at least 3 days per week. These 23 dyads provided assent/consent, completed all baseline assessments, and were randomized to the Generations Health (*n* = 12) or Comparison (*n* = 11) groups. See Fig. 1 for the CONSORT flow diagram.

#### Child participation in the intervention

As far as participation in the Generations Health OOSP sessions, two children opted not to participate in the sessions (one each at week 4 and week 8), but the child/parent dyad that withdrew week 8 completed follow-up assessments. Staff queried these children for their reasons why they stopped participating. The children said they stopped participating in the sessions because they did not like exercising for 40 min, and wanted to be with other friends who were at the Club but not enrolled in the intervention.

For the participation in the Generations Health sessions, the mean number of sessions attended was 25 (SD = 7.9, range = 5–34). Although the goal of the project was for children to attend at least 3 days per week, 4 of the 11 weeks the OOSP was closed on some days for holidays. Adjusting for the holidays, participants were considered to meet the attendance goal if they participated in 25 sessions; 10 out of the 11 (91%) children met the participation goal.

#### Family participation in the intervention

Children in the Generations Health group returned tear-off portions of the take home toolkit cards, signed by an adult, to indicate that they participated in the home activity. On average, children returned 11 (SD 8.5, range 0–25) tear-off portions, documenting their family’s participation in up to 33 home activities. This means that, on average, families participated in about one third of the home activities. The percent of families participating in the study informational sessions was 58% (7/12). The percent of families participating in family nights were 100, 80, and 80% for the first, second, and third events, respectively. Across all three family nights, 25 out of 26 family’s ratings (96%) indicated a high to very high likelihood of participating in similar events again.

#### Parent satisfaction

Parents reported high satisfaction (mean rating of 4, on a 1–5 scale) with the intervention overall. Illustrative parent comments noted the intervention was “very informative and a great way to learn along with the kids,” family nights were “a fun way to involve the whole family,” and the take-home activities provided “new ideas and healthy things to try for dinner and snacks.”

#### Participation in the outcome measures

We determined the feasibility of administering the interviewer-administered on-line ASA 24-dietary recall measure. On average, this measure took 34 min to complete. All children completed the ASA 24-dietary recall measure at pretest and 22/23 (96%) completed it at posttest. To help with these interviews, the child’s parent also attended the recall.

Ninety-six percent (22/23) of children completed the pre- and posttest activity monitor (AM) measures. All children began wearing the AM monitors at the pretest and posttest measurement periods, although two children took the AM off before the 7-day wearing time had been completed. During the pretest measures, one child ended AM wearing 1 day early; during the posttest study measures, one child ended the AM wearing 3 days early (over a weekend).

### Behavioral and health outcomes

Table 3 shows the pretest, posttest, and change scores for behavioral and health outcomes in both groups. Our outcome measure for child adiposity, zBMI, was reduced by 0.15 in the Generations Health group, but increased by 0.13 in the comparison condition. Further, changes in the Generations Health group were significantly greater compared to the comparison group for minutes of moderate-to-vigorous physical activity during the intervention program time (IPT MVPA) period. These findings translated into children in the Generations Health group engaging in 9 more minutes of IPT MVPA during a 40-min period compared to baseline and the difference in IPT MVPA between groups was significant (*P* = 0.028). Daily minutes of MVPA during the week (WD MVPA) significantly increased within each group. Other variables changed in the expected direction; children in the Generations group had decreased intake of daily kcals, higher HEI scores, decreased hours of TV/screen time during a 7-day period, and significantly improved knowledge of nutrition compared to children in the comparison group. Counter to the study hypothesis, children in the Generations Health group had lower hours per night of sleep efficiency and more hours of waking after sleep onset compared to children in the comparison group.

**Table 3:** Behavioral and health outcomes

	Generations (treatment group)						Usual OOSP activities (comparison group)						
	Pre	Post	Change (post-pre)	SD (change)	95% CI	*P* value	Pre	Post	Change (post-pre)	SD (change)	95% CI	*P* value	*P* value^a^
Child’s Nutrition Knowledg^e^, mean^b^	8.8	10.1	1.3	1.7	(0.1, 2.4)	0.036	8.9	9.1	0.2	2.1	(− 1.3, 1.7)	0.770	0.216
Dietary intake													
Kcal^c^	2146	2083	− 62.7	692.2	(− 528, 402)	0.77	1650	2017	367	908	(− 243, 976)	0.210	0.227
Healthy Eating Index^c^	54.9	58.1	3.2	16.8	(− 8.1, 14.4)	0.545	52.8	46.4	−6.3	14.9	(− 16.4, 3.7)	0.189	0.175
Child body mass index (BMI)													
BMI^c^	19.6	19.2	− 0.34	0.83	(− 0.89, 0.22)	0.210	19.8	20.5	0.67	2.35	(− 0.91, 2.25)	0.366	0.196
BMI *z*-score^c^	1.22	1.08	− 0.15	0.31	(− 0.35, 0.06)	0.146	1.03	1.16	0.13	0.64	(− 0.30, 0.56)	0.509	0.208
BMI %^c^	82.2	78.3	− 3.9	11.4	(− 11.6, 3.8)	0.286	73.9	76.9	3.0	12.3	(− 5.3 11.2)	0.445	0.192
Child physical activity and sleep													
WD MVPA^d^	79.3	194.5	115.2	34.9	(90.2, 140.2)	< 0.0001	88.2	184.9	96.7	58.4	(54.9, 138.5)	< 0.0005	0.401
IPT MVPA^d^	10.1	22.9	12.8	8.1	(7.0, 18.6)	0.0007	11.6	15.2	3.6	9.0	(− 2.8, 10.1)	0.234	0.028
Sleep efficiency, hours/night^e^	90.6	89.3	− 1.32	4.5	(− 5.11, 2.46)	0.4358	85.4	86.8	1.36	8.7	(− 4.86, 7.58)	0.633	0.442
Wake after sleep onset, hours per night^e^	1.03	1.01	− 0.02	0.59	(− 0.51, 0.47)	0.9323	1.67	1.22	−0.46	1.37	(− 1.44, 0.53)	0.323	0.416
Screen time per day (hours)^b^	4.23	3.32	− 0.91	3.44	(− 3.22, 1.40)	0.402	4.65	7.05	2.40	5.41	(− 1.47, 6.27)	0.194	0.108

*Abbreviations*: *OOSP* out-of-school program, *IPT MVPA* minutes of moderate-to-vigorous physical activity during the intervention program time period, *WD MVPA* daily minutes of moderate and vigorous activity during the week

^a^*P* value reflects the significance in the change scores between the Generations group versus the Usual OOSP activities group based on a two-sample *t* test

^b^For screen time and child’s nutrition knowledge, *n* = 11 for Generations and *n* = 10 for Usual Care groups

^c^For kcal, BMI, and zBMI, and HEI, *n* = 11 for Generations and *n* = 11 for Usual Care groups

^d^For MVPA vars, *n* = 10 for both Generations and Usual Care groups

^e^For sleep efficiency and wake after sleep onset, *n* = 8 for Generations and *n* = 10 for Usual Care Groups

## Discussion

The results of the Generations Health feasibility study are promising. This study demonstrates the feasibility and potential efficacy of using OOSP behavioral health and family activities to improve BMI and nutrition, increase energy expenditure and MVPA time, and reduce TV/screen time (e.g., sedentary activity time) during the intervention in children, 6–9 years old. The study was designed to maximize the collection of process measures to help inform the delivery of a full-scale trial of the intervention.

Integrating the Generations Health sessions into the OOSP setting and designing assessment and intervention components to meet the needs of participants led to highly successful recruitment and participation of children and their parents. Participation rates in the Generations Health sessions were high (91.3%) for children meeting our goal of participating in at least 25 Generations Health sessions during the 11-week study. These data suggest that the intervention components have strong feasibility for keeping children engaged and interested in regularly attending the program. Our overall retention rate of 83% for the Generations Health activities was higher than a 5-month after-school CATCH Kids Club pilot study that reported a 61% retention rate [38], and a 3-year after-school intervention focusing on MVPA that reported a 44% retention rate [37], but slightly lower than a 12-week after-school intervention with a family component that reported an 87% retention/attendance rate [41], and a community-centered (YMCA), family based, obesity prevention pilot study for overweight and obese children that reported a 90% retention rate at 3 months [55].

Two trained project staff implemented the 1-h Generations Health sessions. Sometimes, a tribal college or university student would assist project staff with the sessions. Session activity boxes provided program staff with helpful instructional and management tips and a variety of games and activities to implement. Equipment and resources to support the session activities were provided and an equipment storage area was available. At the project debriefing session, program implementers reported enjoying the training session, and were faced with few implementation challenges. Implementers became more comfortable and confident with their own abilities as the intervention progressed. We had no staff turnover during the study, and very few behavior problems were reported in children participating in the session activities. There were no problems reported with the older children (ages 8–9) becoming bored/restless being in the same program with the slightly younger children (ages 6–7), suggesting the intervention components were well received by all children in our targeted age range (ages 6–9). Having all eligible siblings participate in the intervention sessions seemed to increase participation of the randomized (enrolled) sibling, as well as other participants. Overall, comments from staff indicated that the Generations Health is a good fit in the OOSP program and staff were interested in continuing its implementation.

Unlike other studies [39], transportation was not a major barrier to participate in the program; this finding was not unexpected as children either walked 8 min, or rode in a van, to the intervention site that was near the OOSP. Parents informally reported to project staff that the major barriers to their children participating in the Generations Health sessions were conflicts with other school (e.g., sports) and family events; parents reported similar barriers in another after-school obesity prevention pilot study [55]. Intervention delivery of the parent/family components was successful and parents reported high satisfaction with the take-home activities and evening sessions.

The low number of returned tear-off portions of the home activities may have been due to parent’s lack of understanding of intent of the tear-off portions, or difficulty returning them through their children. Parents reported that the major barrier to participating in the evening sessions were conflicts with competing events such as school sports and music, and parent work schedules.

Our high measurement participation rates may be due, in part, to the text messages that project staff sent to parents reminding them of upcoming measurement dates. Several parents informally commented that these messages helped them remember to schedule and take part in the study measures. The average time it took participants to complete the ASA24 (34 min) is similar to completion times reported in a study assessing the feasibility of using the ASA24 in preschoolers [56]. Our project staff encountered some challenges related to the Microsoft Silverlight plug-in that was used in the ASA24™-2014 version, challenges that have been reported elsewhere [56]. Some participants experienced “freezing” of the ASA24 midway through completion, although this did not result in the loss of any data. It was unclear whether this arose due to problems with Internet connectivity or other issues. More recent versions of the ASA24 (i.e., ASA24™-2016) no longer use the Silverlight plug-in, potentially resolving this issue. Parents attended the diet recall interview with their child. Most parents gave informal positive feedback about the measure, although some commented that the measurement took a long time. Staff commented that some children lost interest in telling the interviewer what they had consumed in the past 24 h and that having parents at the interview and school breakfast and lunch menus was helpful in prompting the child to remember this information. At least four participants regularly split their time between their parents’ houses. This made collecting diet recall information challenging if the parent attending the interview was not the parent with whom the child had spent the previous 24 h with. In the future full-scale trial, project staff could take steps to become more aware of which parents’ house the child will be at the 24 h before the interview (e.g., by asking both parents prior to the interview date) and make a concentrated effort to schedule the interview with the specific parent (e.g., sending follow-up text reminders about the upcoming interview to the specific parent).

As described earlier, most children (i.e., 96%) completed all activity monitoring measures, but two children had an early end to their AM wearing times. Further, one child wore the AM so inconsistently during pretesting that all of the AM data was lost, and another child simply chose to not wear the AM at all during posttest (though the AM was picked up and returned on schedule with the other participants). Finally, one child wore the AM during all phases of AM monitoring, but the AM itself had malfunctioned and all the data was lost. Even though this child had 100% AM wearing compliance, the malfunctioning error was not recognized until the study was completed. In future studies, before each 7-day AM monitoring period begins, project staff will remind parents in person, through a text message, and an informational sheet, about the importance of children wearing the AM monitor the entire 7 days, and what steps to take (e.g., informing project staff immediately) if the AM is removed or lost. Staff could also check each AM mid-way through the 7-day measurement period to make sure the device is functioning properly.

Even in this short 11-week intervention the reduction in zBMI score in the Generations group trended towards the accepted criterion (≥ 0.25) for clinically important reductions in zBMI [57]. We used zBMI as an outcome because it the simplest surrogate measure of percentage loss in fat mass or adiposity [58]. Similar mean pretest to posttest changes in the expected direction were evident among children for daily kcals, HEI-2010 scores, daily minutes of MVPA, and hours of TV/screen time during the past 7 days for children in the Generations Health vs. comparison conditions, respectively. Additionally, activity monitors showed children in the Generations Health group engaged in significantly more minutes of MVPA during the program time period compared to baseline and this intended effect was not seen in the comparison group. These data show that the MVPA component of the intervention can keep children rigorously engaged for 40 min. Thus, this meets 2/3 of their daily PA requirements of 60 min. This amount, when combined with the routine opportunities for PA available at OOSPs, may help children meet or exceed their daily requirement for physical activity [43]. Although the Generations Health sessions had components for improving sleep quality (e.g., participating in bedtime routine games at home and at the OOSP), the comparison group tended to do slightly better than the Generations Health group for sleep quality. These data suggest the intensity of the sleep components were too low, and that the amount and frequency of activities addressing sleep should be increased in future studies.

At the end of the study, children in the Generations Health group had decreased their daily kcal intake and increased their HEI-2010 scores compared to children in the comparison group who increased their kcal intake and decreased their HEI-2010 score. Further, children in the Generations Health group significantly increased their nutrition knowledge scores compared to children in the comparison group; findings that are similar to other, longer programs that show increased knowledge about food [59]. That participants in the Generations Health group made changes to their kcal intake, HEI-2010 scores and nutrition knowledge after only 11 weeks show strong potential for the nutrition component of the intervention.

Limitations of the present study include imprecise estimates of differences due to the small sample size and the confidence intervals are wide, almost covering zero. Child nutrition knowledge was self-reported and may have been subject to recall bias. Although children in the Generations group went to a location that was near the OOSP to participate in the intervention activities, we cannot entirely rule out that information transfer into the control group did not occur. Even though parents received take-home toolkits to improve nutrition and increase PA in their homes, we did not assess these changes in the home environment. Future studies should incorporate parents or staff completing a survey that assesses home nutrition and PA and child TV/screen time that is based on a validated survey to assess home environments for activity and healthy eating in overweight children [60]. Despite these limitations, this study demonstrated that children and their parents can engage in a high intensity intervention that results in indicators of important changes in children’s weight, PA and diet. Data from this study were used to estimate retention and effect sizes for a future, full-scale trial of the Generations Health intervention in OOSPs situated in rural areas with a high proportion of Native Americans.

## Conclusion

These results suggest that this multi-component intervention could be implemented in OOSP settings and engage parents and families in frequent home-based activities that support their child’s health. The wide reach of OOSPs, and their commitment to fitness and health, suggests these sites are natural partners for the OOSP and home-based childhood obesity prevention intervention. Based on these findings, we will be able to implement a fully powered study with fewer problems and better test the effectiveness of the intervention. If effective, the wide reach of OOSPs suggests strong potential for broad impact on children’s health.
